# Combination chemotherapy with or without s.c. IL-2 and IFN-α: results of a prospectively randomized trial of the Cooperative Advanced Malignant Melanoma Chemoimmunotherapy Group (ACIMM)

**DOI:** 10.1038/sj.bjc.6600043

**Published:** 2002-01-21

**Authors:** J Atzpodien, K Neuber, D Kamanabrou, M Fluck, E B Bröcker, C Neumann, T M Rünger, G Schuler, P von den Driesch, I Müller, E Paul, T Patzelt, M Reitz

**Affiliations:** European Institute for Tumor Immunology and Prevention (EUTIP), Gotenstr. 152, 53175 Bonn, Germany; Medizinische Hochschule Hannover, Germany; Universitätshautklinik Hamburg, Germany; Fachklinik Hornheide an der Universität Münster, Germany; Universitätshautklinik Würzburg, Germany; Universitätshautklinik Göttingen, Germany; Boston University School of Medicine, USA; Universitätshautklinik Erlangen, Germany; Klinikum Nürnberg-Nord, Germany

**Keywords:** melanoma, interleukin-2, interferon alpha, chemotherapy

## Abstract

The purpose of this randomized trial was to evaluate the efficacy of combination chemoimmunotherapy compared with chemotherapy alone. A total of 124 patients were randomized to receive intravenous cisplatin (35 mg m^−2^, days 1–3), carmustine (150 mg m^−2^, day 1, cycles 1 and 3 only), dacarbacine (220 mg m^−2^, days 1–3) and oral tamoxifen (20 mg m^−2^, daily) in combination with (*n*=64) or without (*n*=60) sequential subcutaneous IL-2 and IFN-α. In those patients who received sequential immunotherapy, each cycle of chemotherapy was followed by outpatient s.c. IL-2 (10×10^6^ IU m^−2^, days 3–5, week 4; 5×10^6^ IU m^−2^, days 1, 3, 5, week 5) and s.c. IFN-α (5×10^6^ IU m^−2^, day 1, week 4; days 1, 3, 5, week 5). The overall response rate of patients treated with the combination of chemotherapy and IL-2/IFN-α was 34.3% with seven complete responses (10.9%) and 15 partial responses (23.4%). In patients treated with chemotherapy, only, the overall response rate was 29.9% with eight complete responses (13.3%) and 10 partial responses (16.6%). There was no significant difference in median progression free survival (0 months *vs* 4 months) and in median overall survival (12 months *vs* 13 months) for combined chemoimmunotherapy and for chemotherapy, respectively.

*British Journal of Cancer* (2002) **86**, 179–184. DOI: 10.1038/sj/bjc/6600043 www.bjcancer.com

© 2002 The Cancer Research Campaign

## 

Metastatic melanoma responds poorly to the currently available chemotherapeutic agents and, until today, no standard treatment has been established. Most single agent chemotherapeutics provide only low response rates. Dacarbazine (DTIC), the most active single agent in the treatment of melanoma, can be expected to yield a response rate of about 20% (McClay and McClay, 1996). Even though most trials of combination chemotherapy for the treatment of metastatic melanoma clearly have not been superior to DTIC alone, the combination of carmustine (BCNU), DTIC, cisplatin, and tamoxifen has produced significant antitumour responses in up to 50% of patients (McClay and McClay, 1994; Middleton *et al*, 2000; Richards *et al*, 1992). A further increase in the rate of objective responses may be achieved by introducing cytokines into the therapeutic regimens, with interleukin-2 (IL-2) and interferon alpha (IFN-α) being the most widely acclaimed. Monotherapy with IL-2 results in up to 29% objective responses (Rosenberg *et al*, 1994). Different types of interferons have also been intensively explored (Creagan *et al*, 1990). While a defined subgroup of patients has responded, response rates with immunochemotherapies have been lower than with the best chemotherapy regimens, however, sustained responses have been consistently observed following IL-2 based chemoimmunotherapy.

Recently, several trials have investigated the combination of various cytotoxic agents with IL-2 and IFN-α. While primarily in phase II studies several authors claimed a clear advantage of this chemoimmunotherapy over chemotherapy alone (Legha *et al*, 1996; Richards *et al*, 1992), more recently randomized trials raised doubts about the effectiveness and benefit of this combination (Johnston *et al*, 1998; Rosenberg *et al*, 1999).

We have performed a prospective randomized trial in patients with metastatic melanoma treated either with a chemotherapy regimen of cisplatin, carmustine, dacarbazine, and tamoxifen, or with the identical chemotherapy followed by outpatient s.c. IL-2 and s.c. IFN-α.

## PATIENTS AND METHODS

### Patients

Between May 1995 and May 1999, 124 patients were randomized and considered evaluable for response (Table 1

**Table 1:**
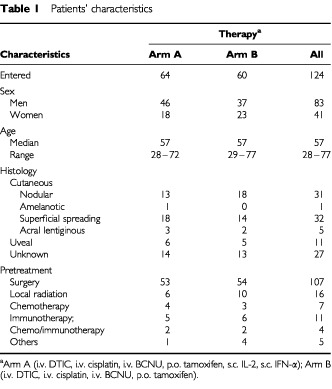
Patients' characteristics

). Forty-one females and 83 males were treated either with combined chemoimmuno- or chemotherapy alone. Median follow-up of these patients was 12 months (range 0–62 months). Ninety-seven patients had not been treated with systemic therapy before, 27 patients had received previous systemic immuno- or chemotherapy treatment.

Criteria for entry into the study were: histologically confirmed metastatic melanoma; Karnofsky performance status >80%; white blood cell count >3500 μl^−1^; platelet count >100 000 μl^−1^; haematocrit >30%; serum creatinin and bilirubin <1.5 of the upper normal limit; age between 18 and 75 years and a life expectancy of >3 months.

This study was approved by the institutional review board of the Medizinische Hochschule Hannover and by participating centres; written informed consent was obtained from all patients prior to entry into the study.

### Study design

All patients received a chemotherapy regimen comprising cisplatin (35 mg m^−2^, i.v., days 1–3), carmustine (150 mg m^−2^, i.v., day 1, cycles 1 and 3 only), dacarbacine (220 mg m^−2^, i.v., days 1–3) and oral tamoxifen (20 mg m^−2^, daily) in combination with (*n*=64) or without (*n*=60) subcutaneous IL-2 and IFN-α. In those patients, who were randomized to receive chemoimmunotherapy, each cycle of chemotherapy was followed by outpatient immunotherapy with combined IL-2 (10×10^6^ IU m^−2^, days 3–5, week 4; 5×10^6^ IU m^−2^, days 1, 3, 5, week 5) and IFN-α (5×10^6^ IU m^−2^, day 1, week 4; days 1, 3, 5, week 5); randomization was performed centrally. Five week treatment cycles were repeated unless progression of disease occurred. Re-evaluation of the patients' tumour status was performed between treatment cycles. Patients received an average of 2.7 (Arm A, median 2.0; range 1–5) and 1.9 (Arm B, median 2.0; range 0–4) treatment cycles.

### Assessment of response

Response to therapy was evaluated according to World Health Organization (WHO) criteria with regular re-evaluation intervals every 8 weeks; all repeated scans were reported to the data center: complete response – disappearance of all signs of disease for a minimum of 8 weeks; partial response – 50% or more reduction in the sum of products of the greatest perpendicular diameters of measurable lesions, no increase in lesion size and no new lesions; stable disease – less than a partial response with no disease progression for at least 8 weeks; progressive disease – 25% or more increase in sum of products in the longest perpendicular diameters of measurable lesions or the development of new lesions. Duration of response was measured from the initial date of response. Two patients were randomized, but did not receive therapy and were evaluated as progressive disease patients. Treatment efficacy was assessed on intent-to-treat basis.

### Statistical analysis

Survival was measured from start of therapy to date of death or to the last known date to be alive. The statistical end points in our analysis were (1) objective response and (2) overall and progression free survival of patients. The progression free survival after 1 year was assumed to be 40% (chemoimmunotherapy) and 20% (chemotherapy), respectively. The intention was to find a chemoimmunotherapy induced progression free survival benefit over chemotherapy alone at 20% with a probability of 95% (α=0.05) and a sample power of 1-ß=0.80. Based on this assumption an intended patient accrual of 64 patients per therapy arm was required in case of a statistically significant survival benefit after combined chemoimmunotherapy. The probability of overall survival was plotted over time according to the method of Kaplan and Meier. Statistical significance was assessed using the Log Rank, Tarone Ware and the Breslow test; SPSS software was employed.

## RESULTS

A total of 124 patients were enrolled in this clinical trial and randomized to receive combined chemoimmunotherapy (*n*=64) or chemotherapy alone (*n*=60) (Table 1). Patient evaluation was performed according to the guidelines described above.

Response to therapy was evaluated according to World Health Organization (WHO) criteria with regular reevaluation intervals every 8 weeks.

### Treatment response

Seven patients (10.9%) treated with chemoimmunotherapy achieved a complete response and 15 patients (23.4%) had a partial remission (Table 2

**Table 2:**
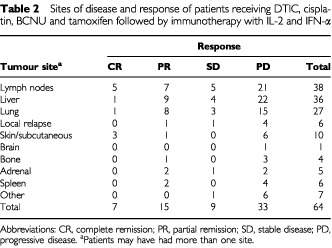
Sites of disease and response of patients receiving DTIC, cisplatin, BCNU and tamoxifen followed by immunotherapy with IL-2 and IFN-α

). The overall objective response rate of combined chemoimmunotherapy was 34.3% (median survival 29 months; 95% CI, 9, 49) with a median duration of response of 9 months (range 5–62 months). While in complete responders, the median response duration was 25 months (range 5–62 months), the median duration of partial responses was 7 months (range 5–19 months). Objective tumour regressions were seen in lymph nodes (12), liver (10), lung (9), skin (4), bone (1), adrenal (2) and spleen (2). Nine patients (14.1%) showed disease stabilization and 33 patients (51.6%) exhibited continuous disease progression despite therapy.

In the chemotherapy group the overall objective response rate was 29.9% (median survival 24 months; 95% CI, 16, 32) with eight patients (13.3%) achieving a complete response and 10 patients (16.6%) a partial remission (Table 3

**Table 3:**
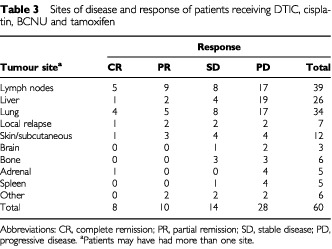
Sites of disease and response of patients receiving DTIC, cisplatin, BCNU and tamoxifen

). The median duration of response ranged from 5 to 47 months (CR, PR, median 15 months). While in complete responders, the median duration of response ranged from 5 to 47 months (median 28 months), the median duration of response in partial responders ranged from 5 to 34 months (median 7 months), respectively. Objective tumour regressions were seen in lymph nodes (14), liver (3), lung (9), skin (4) and adrenal (1). Fourteen patients (23.3%) showed disease stabilization. In 28 patients (46.7%) progression of disease occurred.

### Overall survival

**Figure 1:**
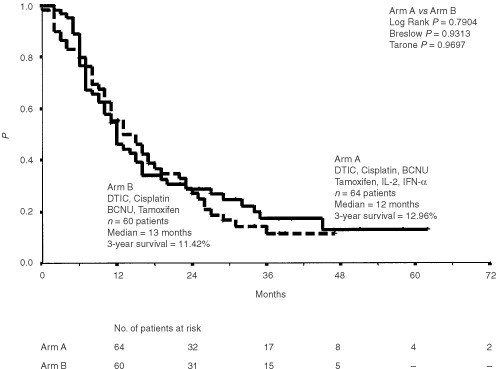
Overall survival (Kaplan-Meier estimates) of 124 patients treated with chemoimmunotherapy (i.v. DTIC, i.v. cisplatin, i.v. BCNU, p.o. tamoxifen, s.c. interleukin-2 and s.c. interferon-α) or chemotherapy (i.v. DTIC, i.v. cisplatin, i.v. BCNU and p.o. tamoxifen) alone. Survival was measured from start of therapy.

**Figure 2:**
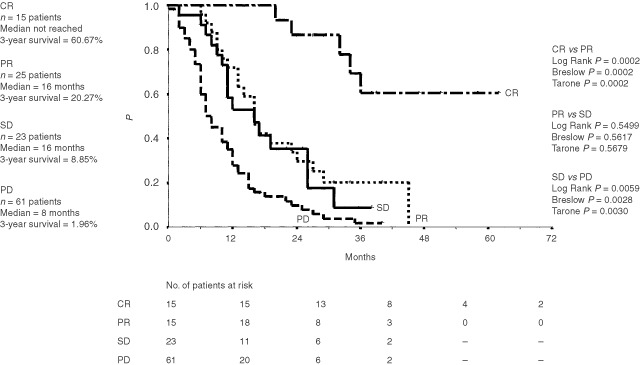
Overall survival (Kaplan-Meier estimates) of 124 patients classified by treatment response. CR=complete remission, PR=partial remission, SD=stable disease, PD=progressive disease. Survival was measured from start of therapy.

Overall median survival of all patients entered into the study was 13 months (95% CI 10; 16). There was no significant difference in median overall survival for combined chemoimmunotherapy (12 months) and for chemotherapy (13 months), respectively (Figure 1

). Patients' sex had also no influence on median survival (data not shown). Twenty-five patients are alive at a median follow-up of 24 months (range 2–62 months). Two patients continue to be alive after more than 5 years from chemoimmunotherapy. A 3-year survival of 12.96% and 11.42% was achieved with combined chemoimmunotherapy and with chemotherapy alone, respectively. For complete responders, median survival has not been reached at 62 months (range 20–62 months). In contrast, partial responders and patients with disease stabilization had both a median survival of 16 months (range 5–40 months and 2–38 months) as opposed to a median survival of 8 months (range 0–40 months) in progressive disease patients (Figure 2

).

In the chemoimmunotherapy group, median overall survival of objective responses was 29 months (range 5–62 months, 95% CI 9, 49), while in the chemotherapy group a median survival of 24 months (range 8–47 months, 95% CI 16, 32) was achieved (data not shown).

**Figure 3:**
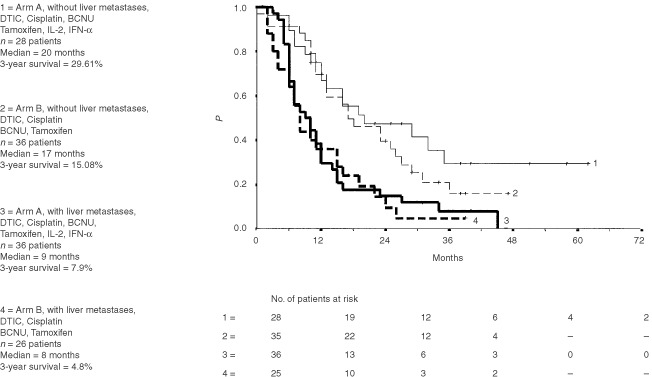
Overall survival (Kaplan-Meier estimates) of 124 patients classified by risk (liver metastases). Survival was measured from start of therapy.

There was a significant (*P*<0.0001) difference in overall survival for all patients with liver metastases with a median survival of 9 months (chemoimmunotherapy) and 8 months (chemotherapy) as opposed to patients without liver metastases with a median survival of 20 months (chemoimmunotherapy) and 17 months (chemotherapy), respectively (Figure 3

).

### Progression free survival

**Figure 4:**
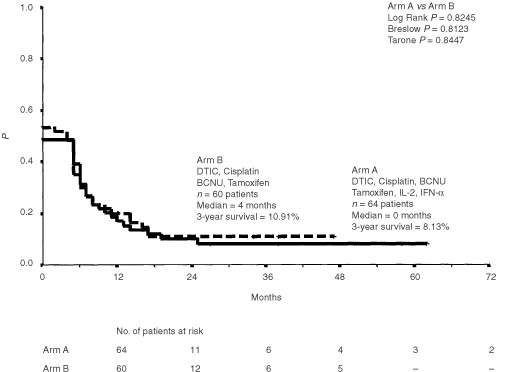
Progression free survival for all 124 patients treated with chemoimmunotherapy (i.v. DTIC, i.v. cisplatin, i.v. BCNU, p.o. tamoxifen, s.c. interleukin-2 and s.c. interferon-α) or chemotherapy (i.v. DTIC, i.v. cisplatin, i.v. BCNU and i.v. tamoxifen) alone. Plots were generated by the Kaplan-Meier method and progression free survival was measured from start of therapy.

The progression free survival ranged from 0 to 62 (median 4 months) for all patients entered into the study. There was no significant difference in median progression free survival between patients treated with chemoimmunotherapy (0 months, range 0–62 months) or with chemotherapy (4 months, range 0–47 months) (Figure 4

).

The median progression free survival for patients with liver metastases was 0 months independent of the treatment regimen as compared to a median progression free survival of 7 months (range 0–62 months, chemoimmunotherapy) and 5 months (range 0–47 months, chemotherapy) in patients without liver metastases (data not shown).

### Treatment toxicity

Chemotherapy led to significant haematotoxicity with WHO grade IV thrombocytopenia in 25% and leukopenia in approximately 26% of treatment cycles, respectively. Immunotherapy related side effects included WHO grade III and IV malaise in 21%, anorexia in 21%, chills in 8%, diarrhoea in 5% and fevers in less than 25% of treatment cycles, respectively (data not shown).

Overall, no life threatening toxicities were observed, and no toxic deaths occurred. Generally, immunotherapy could be administered in the outpatient setting. Chemotherapy required inpatient stays of 4 days per cycle.

## DISCUSSION

In the present prospective randomized trial we reported the results of 124 patients with progressive metastatic melanoma who received a combined chemoimmunotherapy (IFN-, IL-2 combined with DTIC, cisplatin, BCNU, and tamoxifen) or a chemotherapy (DTIC, cisplatin, BCNU, and tamoxifen) alone. We observed an overall objective response in 34.3% of patients treated with the combined chemoimmunotherapy which is comparable with earlier trials achieving response rates between 23% to 64% (Atkins *et al*, 1994; Atzpodien *et al*, 1995; Feun *et al*, 1995; Hoffmann *et al*, 1998; Johnston *et al*, 1998; Legha, 1998; Middleton *et al*, 2000; Pyrhonen *et al*, 1992; Rosenberg *et al*, 1999; Thompson *et al*, 1997). Whereas in previous clinical trials, introducing cytokines in chemotherapeutic regimens yielded an enhanced efficacy (Atkins *et al*, 1994; Legha *et al*, 1996), our results raise doubts concerning the potential benefits of the presently used dosages of IL-2 and INF-α for therapy of metastatic melanoma since objective response rates of patients treated with chemotherapy alone were similar (29.9%) to sequential chemoimmunotherapy (34.3%).

In addition, in our trials no significant difference in survival time was observed for patients treated with chemoimmunotherapy (median 12 months) and chemotherapy (median 13 months), respectively, which is also supported by recent results of other cytokine based therapy regimens (Johnston *et al*, 1998; Rosenberg *et al*, 1999). Although overall survival of objective responses was slightly increased in the chemoimmunotherapy group (median 29 months, 95% CI 9, 49) than in the chemotherapy group (median 24 months, 95% CI 16, 32), the response duration of patients was shorter after chemoimmunotherapy (median 9 months) when compared to chemotherapy (15 months). In contrast, Legha *et al* (1997) reported a significantly increased proportion (10%) of long-term responders after chemoimmunotherapy.

The discrepancy in the effect of cytokines in metastatic melanoma therapies might be best explained by the following uncontrolled variables: (A) patient selection and clinical/biological risk; (B) variations in the rIL-2 based regimen (e.g., reconstitution of drug, route and dose of administration); and (C) different standards of patient care (e.g., supportive care upon IL-2 therapy). In fact, previous reports have repeatedly shown that overall survival is higly risk-dependent (Hoffmann *et al*, 1998; Rusthoven *et al*, 1996). This is confirmed by our study since we reported that patients with liver metastases had a significantly shorter survival and shorter progression free survival compared to patients without liver metastases. Notably, the proportion of patients with hepatic metastases varied between 56% (chemoimmunotherapy) and 43% (chemotherapy alone).

In summary, this prospectively randomized clinical trial showed no statistically significant benefit of IL-2, INF- based combined chemoimmunotherapy in patients with metastatic melanoma compared to chemotherapy alone. So far, also no standard was established confirming a significant survival benefit of polychemotherapy when compared to DTIC, alone (Chiarion Sileni *et al*, 2001).

While median duration of objective responses appeared to be prolonged in patients receiving chemotherapy only, overall survival analysis yielded few (*n*=2) long term (>48 months) surviving patients in the chemoimmunotherapy combination, only. Similar results were reported when comparing dacarbazine/INF- with or without IL-2. Since cytokines in chemotherapies also alter the toxicity profile (Falkson *et al*, 1998; Johnston *et al*, 1998; Stark *et al*, 1998), future clinical trials might focus on new combinations, different dosages and dose distribution regimens of IL-2 and INF- to increase both efficacy and tolerability for patients with metastatic melanoma.
